# A Novel Plasma Microscalpel Based on Airplasma® Technology. A Histological Analysis of Brain Tissue Damage

**DOI:** 10.1177/15533506261437503

**Published:** 2026-04-20

**Authors:** Francesco Guerrini, Alessandra Viglio, Cesare Zoia, Valeria Musella, Pietro Serinelli, Catherine Klersy, Pietro Zangrossi, Viola Marta Custodi, Erika Ferrari, Giuseppe Bellantoni, Francesco Salomi, Giannantonio Spena

**Affiliations:** 1 Unit of Neurosurgery, Department of Head & Neck Surgery, 18631 Fondazione IRCCS Policlinico San Matteo, Pavia, Italy; 2 Department of Internal Medicine, University of Pavia, Italy; 3 Unit of Pathology, Department of Diagnostic and Laboratory Medicine, 18631 Fondazione IRCCS Policlinico San Matteo, Pavia, Italy; 4 Unit of Neurosurgery, 89497 Moriggia-Pelascini Hospital, Gravedona, Italy; 5 Unit of Biostatistics and Clinical Trial Center, Fondazione Policlinico IRCCS San Matteo, Pavia, Italy; 6 Department of Neurosurgery, Sant’Anna University Hospital and Department of Translational Medicine, University of Ferrara, Ferrara, Italy

**Keywords:** plasma, airplasma, brain tissue damage, lateral thermal injury, microscalpel, monopolar cautery, bipolar cautery

## Abstract

**Background:**

Microscalpel based upon Airplasma® technology showed promising results in dermatology, plastic surgery and veterinary in terms of tissue lateral thermal damage (LTI). In this preclinical work, we aimed to explore its effect on central nervous system tissues which has not been established yet.

**Methods:**

Samples of glioma and meningioma tissue were cut by plasma microscalpel (PM) and bipolar forceps (BC). A pathologist, blinded to the assignment of samples, analyzed the presence of histological damage, that was the primary endpoint. This was a composite of 4 pathological aspects. A comparison between different types of cutting was performed.

**Results:**

Forty-three patients were included in the final analysis. Histological damage was observed in 14.29% of PM samples and in 46.34% of BC ones (*P* 0.001). A sensitivity analysis of the primary endpoint, based on significant damage in each pathological aspect, revealed significantly better results for PM samples.

**Conclusions:**

Microscalpel based on Airplasma® technology showed less cellular damage if it is compared to BC. Even if it cannot supplant the bipolar cautery, this instrument could be important in microneurosurgery in terms of precise cutting, minimized temperature, very low lateral LTI and the possibility to reset mechanical forces.

## Introduction

The Airplasma® technology functions by ionizing atmospheric air to generate plasma, thereby serving as an efficient energy carrier. This process is based on negating the insulating properties of air through exposure to a high-voltage, oscillating electromagnetic field. The applied field disrupts dielectric bonds, effectively converting air into a conductive medium. The resultant plasma emission manifests as a visible luminous glow. Consequently, the cutting process occurs at a low temperature (<50°C) without the need for additional mechanical force.^1^ Hemostasis is achieved through protein denaturation, facilitating gentle coagulation of capillary vessels. This technology has been already implemented in various surgical fields, including dermatology, plastic surgery, and veterinary medicine; however, its application in microneurosurgery remains unexplored.^1-5^

In this interventional preclinical study, we describe our experience using a microscalpel based on airplasma technology (Oneyonis® A 1000, Otech Industry, Alessandria, Italy) to evaluate its tissue effects on pathological examination, particularly in terms of lateral thermal injury (LTI).

## Materials and Methods

Patients over 18 years old treated at Fondazione IRCCS Policlinico San Matteo from November 2023 onward for newly diagnosed intracranial glioma or meningioma were included in the study.

### Device Description and Peculiarities

The Plasma microscalpel (PM) is a plasma thermo-coagulation electrosurgical ablation device. It is composed of a central unit equipped with intensity control settings ranging from 0 to 100 and another knob for pulse setting; a handpiece sterilizable in an autoclave, a needle-shaped tip (straight tip electrode Ø 0.3 mm, length 20 mm), and a foot pedal for activation. The initial plasma generation occurs by holding the handpiece electrode without direct contact with the tissue, maintaining an air gap ranging from a few tenths of a millimeter up to 3 mm, depending on the applied power. This gap is essential for the potential difference to initiate air ionization. Subsequently, as soon as the electrode comes into contact with the tissue, the cutting or dissection process begins. The pulse setting modulates the continuity of plasma emission for adjustable durations, albeit within fractions of a second. This function is indicated in delicate areas, where maintaining a stable plasma output is crucial while simultaneously reducing tissue impact through intermittent energy delivery.

### Device Description and Peculiarities

The Plasma microscalpel (PM) is a plasma thermo-coagulation electrosurgical ablation device. It is composed of a central unit equipped with intensity control settings ranging from 0 to 100 and another knob for pulse setting; a handpiece sterilizable in an autoclave, a needle-shaped tip (straight tip electrode Ø 0.3 mm, length 20 mm), and a foot pedal for activation. The output voltage is 2200 Volt; the current density during operation is 0.050 A; the electrical power absorbed/delivered (in watts) is 75VA (maximum absorbed power) and 13 W (maximum output power). Waveform characteristics are: frequency 50 Hz, duty cycle 60% and no modulation.

The initial plasma generation occurs by holding the handpiece electrode without direct contact with the tissue, maintaining an air gap ranging from a few tenths of a millimeter up to 3 mm, depending on the applied power. This gap is essential for the potential difference to initiate air ionization. Subsequently, as soon as the electrode comes into contact with the tissue, the cutting or dissection process begins. The pulse setting modulates the continuity of plasma emission for adjustable durations, albeit within fractions of a second. This function is indicated in delicate areas, where maintaining a stable plasma output is crucial while simultaneously reducing tissue impact through intermittent energy delivery.

### Workflow

Following tumor excision, the specimen was divided into 3 sections: one allocated for diagnostic evaluation in accordance with the 2021 WHO classification and 2 experimental samples, designated as A and B, measuring a diameter of about 1 cm. Sample A underwent incision on all 4 sides using a PM, while sample B was subjected to coagulation along all 4 edges via bipolar cautery (BC) (VIO 200 D, Erbe Elektromedizin, Tübingen, Germany). The application duration for both instruments was standardized at approximately 10 seconds per surface. Subsequently, all samples were fixed in 10% formalin and submitted for histopathological analysis within 24 hours since withdraw. Pathologist was blinded to the treatment method assigned to samples A and B (plasma incision or bipolar cauterization).

Bipolar cautery was selected as the comparator due to its routine use as the sole electrocautery modality in microneurosurgery as it guarantees the most focused and safe cauterization. Monopolar cautery instead was excluded as a comparator because it is not routinely used in microneurosurgery due to the highest operational temperatures. On the other hand, comparison with a cold blade was deemed inappropriate for histological analysis of thermal injury (LTI), as cold incisions, by definition, do not induce thermal damage.

PM was set at 50% power with continuous waveforms, which enables a strong cutting power; BC was set at 50 W, effect 3.

### Histological Analysis

After measurement and macroscopic description, samples were embedded in paraffin and sectioned into microslices 5-6 μm thick using a microtome. Hematoxylin-eosin staining was performed. A pathologist, blinded to the assignment of samples A and B, examined the samples for the following elements:

• Coagulative necrosis or eosinophilic condensation
• Nuclear elongation with unidentifiable morphology
• Homogeneous, eosinophilic cytoplasm
• Shrinkage effect, with nucleus-cytoplasm separation

The pathologist quantified these features as a percentage of all analyzed sections. A threshold of 10% was chosen to define significant lesions. Each pathological feature was assessed on a binary basis (present or absent) for each histological section. No semi-quantitative grading scale for severity or extent was applied. For each sample, the number of sections showing a given feature was divided by the total number of analyzed sections, yielding the percentage of involvement for that feature. A predefined threshold of ≥10% of analyzed sections was used to define significant damage.

### Endpoints

The primary endpoint was the microscopic damage, defined by the presence of at least 3 of the aforementioned elements, compared between samples A and B. This composite endpoint was chosen to capture the multifactorial nature of thermal-induced cellular injury in neural tissue, rather than relying on a single histopathological parameter.

The secondary endpoint was the satisfaction score on the Numeric Rating Scale (NRS), ranging from 0 (not satisfied) to 5 (completely satisfied), concerning the ergonomics and usability of the plasma cautery. The following aspects were evaluated: ease of machine and accessory installation, output power setting regulation, and scalpel handle ergonomics. All the procedures were effectuated by 3 different surgeons (GS, FG, SF).

### Sample Size

Based on feasibility, approximately 40 subjects will be enrolled, each providing one sample for a total of 40 samples. With 40 samples, it will be possible to highlight an absolute difference in the proportion of cuts with histological lesion (primary endpoint) of −30% with power greater than 80%, assuming a proportion of 70% in the cut with bipolar coagulator and an intra-sample correlation of at least 30% and a two-tailed alpha error of 5%. The calculation is based on McNemar’s test for paired samples.

### Statistical Analysis

Continuous data were described with the mean and standard deviation (SD) or the median and 25^th^-75^th^ percentiles if skewed, categorical variables as counts and percent. For the primary endpoint, proportions of tissue damage were compared using a generalized linear regression model within the binomial family. Huber-White robust standard errors were calculated to account for intra-subject aggregation. The model estimated proportion differences and 95% confidence intervals (95%CI). Sensitivity analysis of the primary endpoint was conducted using the same model across 4 levels of pathology effects. A generalized linear regression model was applied to compare the 2 approaches. The secondary endpoints are only descriptive and proportion of times a satisfaction score of 5 is reported.

A 2-sided *P*-value <0.05 was considered statistically significant. The Stata software (release 18.5, StataCorp, College Station, TX, USA) was used for computation.

This is a profit study was approved by the local Ethical Committee (v 2022-3.11/653) sponsored by Otech Industry, Alessandria, Italy.

## Results

Forty-nine patients were enrolled in the study, but 6 cases were excluded due to the inability to collect valid samples (insufficient size or too soft consistency that made impossible to precisely cut the sample). Ultimately, 43 patients were included in the analysis. The mean age was 60.4 years (SD 14), and the male-to-female ratio was 0.83. Pathological examination revealed a glial lesion in 24 cases, while the remaining patients had meningiomas.

### Assessment of the Histologic Damage (Primary Endpoint)

The results of the study are summarized in Table 1. Overall, the histologic damage was significantly lower in PM cuttings at 14% of PM as compared to 46% of BC cuttings (Difference 32% (95% CI 13-51, *P* = 0.001). Consistently, the analysis of the components of the primary endpoint showed higher percentages of specimen with damage in the BC for all features, with the highest differences observed for the homogeneous cytoplasm feature (difference 39% (95% CI 11-39, *P* < 0.001) and the nuclear elongation feature (difference 37% (95% CI 19-55, *P* < 0.001). These data corresponds to significantly higher percent of damage of the analyzed sections in the BC cuttings (Figure 1).

**Table 1.** Comparison of Cutting Approaches

Endpoint	PM	BC	Mean difference (95% CI)*	*P*-value
Primary endpoint				
Histologic damage (N, %)	6 (14%)	19 (46%)	−32% (−51 to −13)	0.001
Sensitivity analysis of the primary endpoint				
Coagulative necrosis or eosinophilic condensation				
• Above cut-off (N, %)	6 (14%)	12 (29%)	−15% (−31 to 1)	0.060
• Percent of analysed section (mean, SD)	5.7 (8.7)	15.2 (16.2)	10 (5-14)	<0.001
Nuclear elongation with unidentifiable morphology				
• Above cut-off (N, %)	7 (17%)	22 (54%)	−37% (−55 to −19)	<0.001
• Percent of analysed section (mean, SD)	7.7 (7.8)	20.7 (16.5)	13 (8-18)	<0.001
Homogeneous, eosinophilic cytoplasm				
• Above cut-off (N, %)	6 (14%)	22 (54%)	−39% (−58 to −21)	<0.001
• Percent of analysed section (mean, SD)	8.0 (7.2)	22.3 (18.3)	14 (8-20)	<0.001
Shrinkage effect, with nucleus-cytoplasm separation				
• Above cut-off (N, %)	8 (19%)	20 (49%)	−30% (−49 to−11)	0.002
• Percent of analysed section (mean, SD)	8.0 (7.4)	18.4 (13.4)	10 (6-14)	<0.001
Secondary endpoints: Rate of NRS = 5		//	//	//
Ease of machine installation (N, %)	42 (100%)	//	//	//
Ease of accessory installation (N, %)	42 (100%)	//	//	//
Output Power setting regulation (N, %)	40 (97%)	//	//	//
Scalpel Handle ergonomics (N, %)	29 (69%)	//	//	//

Abbreviations: PM, Plasma microscalpel; BC, bipolar cautery. *Estimated from model.

**Figure 1.**
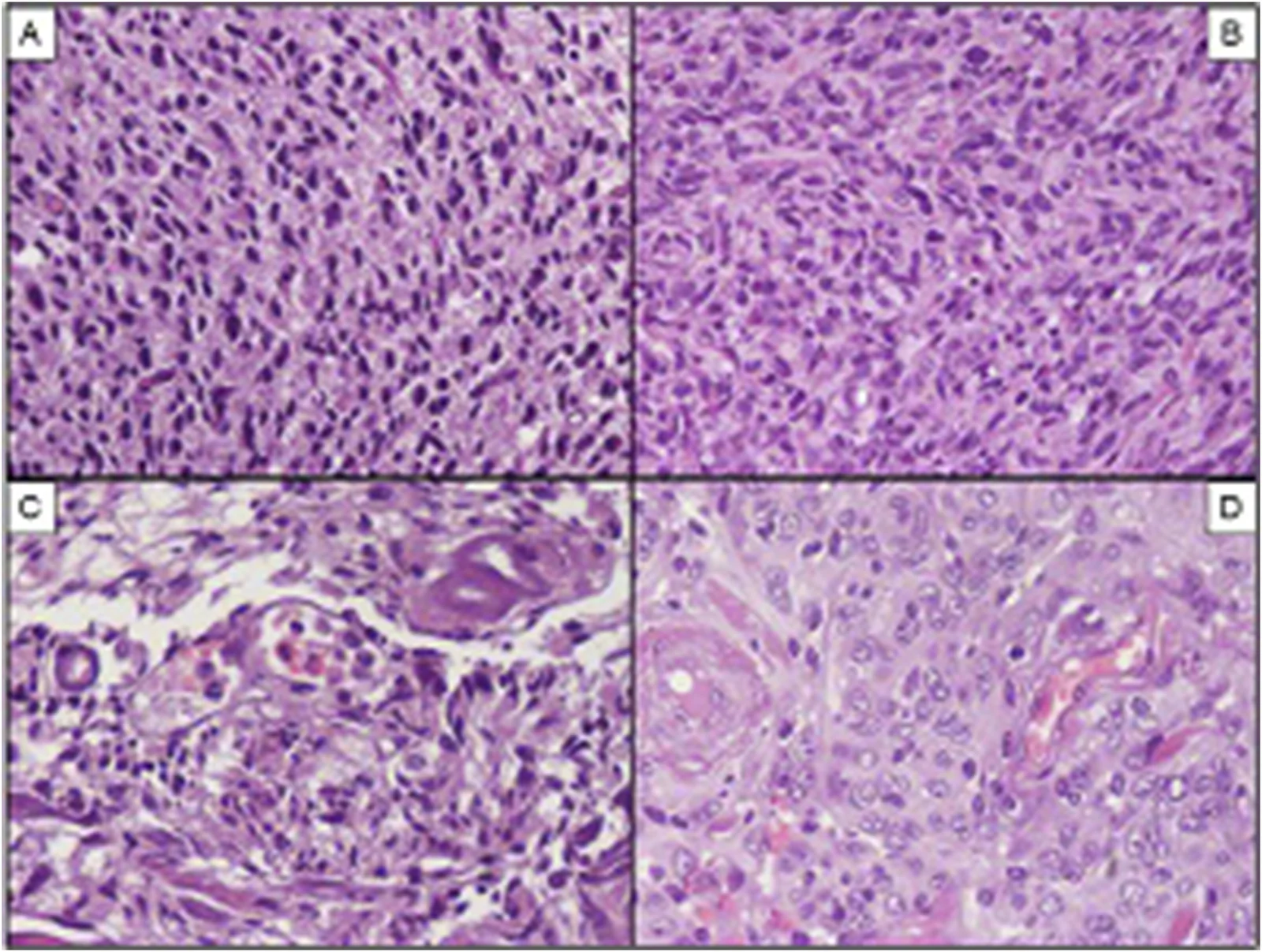
Pathological features of samples cutted with BC and PM. (A) Hematoxylin-eosin (HE), 20x, glioblastoma, BC; C, HE, 20x, meningioma, BC: in both pictures, nuclei are elongated and of unidentifiable morphology and cytoplasms are uniformly eosinophilic. (B) HE, 20x, glioblastoma, PM; D, HE, 20x, meningioma, PM: the same cases of pictures A, C display an excellent cytological detail. BC, bipolar cautery; PM, plasma microscalpel

### Assessment of the Satisfaction Scores (Secondary Endpoints)

As for the secondary endpoints, surgeons’ evaluation of the use of PM in each cutting was at the highest range of 5 of the NRS scale for all aspects of usability of the plasma cautery, while 69% considered the ergonomics as optimal, while the remaining surgeons at a level of 4 (Table 1).

## Discussion

The current study is the first ex vivo experience of the effects of PM on human nervous tissue. We specifically addressed the LTI in terms of cellular damages defined as the presence of at least 3 pathological features. For samples cut with PM, all histological signs of cellular damage were present in a median of 5%, whereas in the control samples these damages were at least doubled. This evidence reached statistical significance showing worse results for all BC samples. This was further confirmed by the sensitivity analysis of the primary endpoint, both in terms of significant damage and raw percentage. These results are likely due to the intrinsic mechanisms of plasma technology, in which air serves as the energy vector. When the tip of the electrode interacts with tissue electrolytes, it generates a highly ionized field, enabling dissection at lower temperatures.^6,7^

Neural tissue lesions following application of electrocautery based devices are in general related to different mechanisms, ischemia being the primary trigger of this process and thermal damage plays an important role as well. The denaturation of proteins leads to disruption of mitochondria and other organelles, giving the cytoplasm a homogeneous eosinophilic appearance. Subsequently, pyknosis, karyorrhexis, and karyolysis occur, leading to cell shrinkage and nuclear elongation.^8^

Dewhirst et al demonstrated that neural tissues, in particular, are highly susceptible to heat, with a low threshold temperature of approximately 43°C. Specifically, peripheral nerves exhibit the highest resistance, while the spinal cord demonstrates intermediate tolerance, with the brain being regarded as the most susceptible to damage from local hyperthermia.^9^ Elliott-Lewis et al showed that bipolar cautery can reach temperatures of 60°C after 5 seconds and 100°C after 10 seconds.^10^

Airplasma technology has been investigated and is currently implemented across various surgical specialties, including general, oral, dermatologic, and oncologic surgery, as a substitute for monopolar cautery in both tissue incision and hemostasis.^11,12^ The plasma discharge is generated through short-duration bursts, high-frequency pulsed, or continuous waveforms, facilitating a less traumatic cutting force, with air serving as the energy vector. Notably, the present PM eliminates the necessity for external sources of inert gases (such as argon or helium) and closed-circuit return electrode systems, as required in technologies like Argon Plasma Coagulation (APC).

Compared with traditional ‘cold’ instruments such as microscalpels, scissors, and dissectors, plasma-based cutting avoids the transmission of mechanical forces to surrounding tissue, which may be particularly relevant when operating near delicate neurovascular structures. Conversely, while cold instruments are intrinsically free of thermal injury, they rely entirely on manual traction and counter-traction during dissection.

From a microneurosurgical standpoint, the primary appeal of this technology could lie in its low operational temperature and high cutting precision, particularly when utilizing an ultrafine tip (0.3 mm). These attributes, combined with the ability to achieve gentle coagulation of arterial and venous capillaries, suggest that the PM probe could serve as a microdissector with the additional capability of precise tissue incision and hemostasis, without requiring mechanical force application. In microneurosurgery, delicate dissection of tumors or other pathological entities from critical neurovascular structures is often necessary. Surgeons typically rely on microscalpels, scissors, and dissectors, while in cases of hemorrhage, bipolar cautery is preferred alongside sponges or other hemostatic agents to minimize collateral damage.

In the context of evolving neurosurgical instrumentation, lasers have also been explored due to their ability to provide fine dissection and coagulation.^13,14^ However, enthusiasm for their use in open neurosurgery has waned due to technical limitations and associated risks. CO_2_ lasers, for instance, require transmission via optical fibers, and their long wavelength restricts efficient energy delivery to the surgical field.^15^ Ryan et al conducted a similar study in which the cortex of 6 anesthetized swines was cutted by CO_2_ laser or BC or n°11 scalpel. Light microscopy revealed a crater zone, then a desiccated tissue and an edematous one. All 3 zones were narrower in comparison with those obtained by BC and authors attributed this finding with a lower LTI in case of CO_2_ laser delivery. Nevertheless, silver staining technique revealed axonal swelling and balloons in desiccated and edematous zones of sample incised by 7 W CO_2_ laser and this is maybe related to operational temperature of this device that exceeds 100°C, increasing the risk of excessive thermal damage.^14^ Similarly, neodymium-doped yttrium aluminum garnet (Nd:YAG) and other lasers, which are frequently integrated into endoscopic systems, have demonstrated potential for damaging healthy brain tissue adjacent to the ablation site.^16-18^

Our study presents some limitations. First, as a preclinical study, it is not yet possible to generalize our results to support the routine use of PM in daily neurosurgical practice. Second, the study design limits our ability to draw conclusions about microvessel coagulation. As a result, we have already planned a clinical study to evaluate the advantages and disadvantages of in vivo Airplasma® application.

Building on the present ex vivo findings, a prospective single-center pilot clinical study is currently being planned to evaluate the in vivo application of the plasma microscalpel in microneurosurgical procedures. The primary endpoint of this study will be safety, assessed through intraoperative and early postoperative complications, with particular attention to unintended thermal injury to adjacent neural or vascular structures. Secondary endpoints will include hemostatic efficacy at the level of microvessel coagulation, precision and controllability of tissue dissection in real surgical scenarios, surgeon-reported usability, and feasibility of integration into standard microneurosurgical workflows. Unlike the present ex vivo model, the in vivo setting will allow evaluation of dynamic factors such as tissue perfusion, bleeding control, and interaction with microvascular structures. Study initiation is anticipated within the next 12-18 months.

## Conclusions

The current experience on the application of Airplasma® technology onto central nervous system tissue demonstrated a substantial reduced cellular harm if compared to the routinely used bipolar electrocautery. This outcome is likely attributed not solely to its capacity for breaking and altering protein bonds, but also to the low operational temperatures (below 50°C). This study was not intended to present the plasma technology as an alternative to bipolar cautery, which is still of paramount utility in neurosurgery. Rather, we can hypothesize that this instrument could be applied to microneurosurgery where precise cutting, minimized temperature, very low lateral LTI and the absence of mechanical forces (like microscissors or cold microblades) are of paramount importance. Further studies are needed to confirm its utility in clinical practice.
